# Effects of Crop Canopies on Rain Splash Detachment

**DOI:** 10.1371/journal.pone.0099717

**Published:** 2014-07-03

**Authors:** Bo Ma, Xiaoling Yu, Fan Ma, Zhanbin Li, Faqi Wu

**Affiliations:** 1 State Key Laboratory of Soil Erosion and Dryland Farming on Loess Plateau, Institute of Soil and Water Conservation, Northwest A&F University, Yangling, Shaanxi Province, China; 2 College of Resources and Environment, Northwest A&F University, Yangling, Shaanxi Province, China; 3 Institute of Desertification Control, Ningxia Academy of Agriculture and Forestry Science, Yinchuan, Ningxia Hui Autonomous Region, China; Centro de Investigacion Cientifica y Educacion Superior de Ensenada, Mexico

## Abstract

Crops are one of the main factors affecting soil erosion in sloping fields. To determine the characteristics of splash erosion under crop canopies, corn, soybean, millet, and winter wheat were collected, and the relationship among splash erosion, rainfall intensity, and throughfall intensity under different crop canopies was analyzed through artificial rainfall experiments. The results showed that, the mean splash detachment rate on the ground surface was 390.12 g/m^2^·h, which was lower by 67.81% than that on bare land. The inhibiting effects of crops on splash erosion increased as the crops grew, and the ability of the four crops to inhibit splash erosion was in the order of winter wheat>corn>soybeans>millet. An increase in rainfall intensity could significantly enhance the occurrence of splash erosion, but the ability of crops to inhibit splash erosion was 13% greater in cases of higher rainfall intensity. The throughfall intensity under crop canopies was positively related to the splash detachment rate, and this relationship was more significant when the rainfall intensity was 40 mm/h. Splash erosion tended to occur intensively in the central row of croplands as the crop grew, and the non-uniformity of splash erosion was substantial, with splash erosion occurring mainly between the rows and in the region directly under the leaf margin. This study has provided a theoretical basis for describing the erosion mechanisms of cropland and for assisting soil erosion prediction as well as irrigation and fertilizer management in cultivated fields.

## Introduction

Soil-particle splashing caused by raindrop impacts on the ground during rainfall is usually evenly distributed if farmlands are not covered with crops. However, crop growth and coverage disturb this uniformity. The course of rainfall through crop canopies can be divided into three parts: throughfall, stemflow, and canopy interception [1]. Among these, throughfall has the strongest influence on soil splash erosion. Studies have confirmed that, due to the wide row spacing of cultivated crops, coverage in the center of the between-row area is usually very low during a large part of the growing season, and that therefore throughfall in this position was significantly greater than in regions closer to the plants. The uniformity of throughfall distribution under a densely planted crop canopy was higher than under intertilled crops [2]. Therefore, emphasis should be placed on intertilled crops when studying the effects of crops on splash erosion.

Rainfall splash erosion is the initial stage of water erosion and occupies a prominent position in the formation and evolution of erosion [3], [4], [5], [6]. The power of splash erosion is related to the size, shape, terminal velocity, and kinetic energy of raindrops. In addition, it is closely related to slope gradient, slope aspect, soil properties, and vegetation cover [7]. On cultivated land, if other conditions were relatively uniform, the biological characteristics of crops would become the main factor affecting splash erosion. Armstrong and Mitchell [8] indicated that in some positions under the crop canopy, throughfall intensity increased considerably compared with rainfall intensity higher in the canopy. The median diameter (D_50_) of rainfall under corn and soybean canopies was larger than that of natural rainfall, and many large-diameter raindrops (≥50 mm) dropped from a height of more than one meter, creating substantial erosion. Although crop canopies can reduce rainfall energy, if vegetation cover at a distance of 0.3 meter from the surface is not yet fully developed or completely canopied, soil detachment caused by large raindrops will still occur [9], [10]. Generally, splash erosion amounts decreased with increasing coverage, and the closer the cover is to the ground, the lower are the splash erosion amounts [11]. Morgan [12], [13] found that splash erosion yields under wheat canopies over one hundred days decreased as the kinetic energy of rainfall increased and that soil protection by crops under high-intensity rainfall was stronger than under low-intensity rainfall. It was also noted that the effects of crops on the number of throughfalling raindrops, the size distribution of raindrops, and raindrop energy characteristics were the major factors affecting splash erosion under canopies. This viewpoint was confirmed by Finney (1984), who observed and analyzed the mean diameter of raindrops, rainfall kinetic energy, and splash erosion yields under the canopies of several vegetable crops [14].

The Loess Plateau in China is one of the areas in the world which is seriously plagued by soil erosion. Sloping land in this region covers 875.97 ha, accounting for 55.69% of total land area. Erosion is the main source of soil and water loss, and erosion yield is approximately 50%–60% of total erosion yield in this region [15], [16]. Therefore, studies of the mechanisms of soil and water loss in sloping fields as well as possible protective measures have become very important. However, in the current research situation, studies of the mechanism of crop protection from erosion are still relatively rare. This study has focused mainly on analyzing the variation and spatial distribution of splash erosion rates under corn, soybean, millet, and winter wheat canopies at different growth stages to reveal the effects of soil splash erosion and to provide a basis for soil erosion prediction based on previous studies.

## Materials and Methods

### Study Site

This study was carried out from 2007 to 2009 at the Soil and Water Conservation Engineering Laboratory, Northwest A&F University (Shaanxi province, P.R. China), situated in the southern fringe of the Chinese Loess Plateau. The exact geographical position is 113.08° East longitude and 34.58° North latitude, with an elevation 468 m above the mean sea level. Soil in the study area, according to the Chinese Soil Taxonomy, is Eum-Orthic Anthrosols, which is a kind of Cinnamon soil [17]. The climate of the study area is semi-humid monsoon. Most precipitation (nearly 60% of total rainfall) typically occurs between July and October, and the annual rainfall ranges from 635 to 646 mm. The mean monthly maximum temperature is 26.1°C in July, and the mean minimum temperature is −1.2°C in January. The main crops in the study area are corn (*Zea mays L.*), soybeans (*Glycine max merr.*), cotton (*Gossypium hirsutum Linn.*), winter wheat (*Triticum aestivum Linn.*), and millet (*Setaria italica Beauv.*).

### General Information

Since 2006, corn, soybeans, millet, and winter wheat have been planted according to their sowing seasons. The corn used in this study was Zhengdan-958, and seeding started on June 20, 2009. According to local conditions, the line and row spacings of corn land are 60 cm and 25 cm. The soybean used in this research was Zhonghuang-13, and seeding began on June 30, 2007, with a planting density of 20 cm×40 cm. The millet used in this study was Jingu-29, and seeding began in 2008 with a planting density of 10 cm (plant spacing) and 20 cm (row spacing). The wheat used in this research was Xiaoyan-22 and was sowed using drill seeding with a seeding quantity of 130 kilograms per hectare. Planting management was conducted according to local customs. The soil used in the study was Eum-Orthic Anthrosols, and rainfall intensities were 40 mm/h and 80 mm/h, with 30 minutes of rain at one time according to the characteristics of local storms which are concentrated in summer and autumn. The crop growth, vegetative growth stages and average leaf area for each sample time are shown in table 1.

**Table 1:** Crop growth and vegetative stage at each sampling date.

Crops	Observing date	Growth stage	Symbol	Average plant height (cm)	Leaf area(cm^2^ plant^−1^)	LAI
**Corn**	2009/7/10	Seedling stage	V4	35	470	0.31
	2009/7/25	Early jointing stage	V6	92	2220	1.48
	2009/8/3	Middle jointing stage	V9	128	4250	2.83
	2009/8/10	Late jointing stage	V12	161	4830	3.22
	2009/8/17	Tasseling stage	VT	215	6470	4.31
**Soybean**	2007/7/30	Initial blossoming stage	R1	38	1730	2.16
	2007/8/10	Full flowering stage	R2	46	3020	3.77
	2007/8/20	Initial pod-filling stage	R4	76	4170	5.21
	2007/8/28	Pod-bearing stage	R6	79	5210	6.51
**Millet**	2008/5/25	Fifth leaf stage	GS2	43	170	0.86
	2008/6/6	Flag leaf visible stage	GS4	68	310	1.54
	2008/6/19	50% stigma emergence	GS6	85	440	2.18
	2008/6/29	Milk stage	GS7	112	620	3.11
**Winter wheat**	2008/3/15	Stem elongation stage	Feekes 6.0	16	50	2.32
	2008/4/1	Jointing stage	Feekes 9.0	38	70	3.61
	2008/4/15	Early heading stage	Feekes 10.1	52	100	4.82
	2008/5/2	Anthesis flowering stage	Feekes 10.52	86	120	6.12

If the one digit was 5 or greater, rounded to the greater ten in column of leaf area.

The rainfall simulator in this study was designed and constructed by the Institute of Soil and Water Conservation, Yangling, China. For indoor rainfall simulation, the downward-facing sprinkling rainfall simulation system was similar to that used by Jin *et al.* [18]. Four nozzles were positioned at a drop fall height of 4 m. The rainfall simulator consisted of two 3 m-long sprinkler booms, positioned at a distance of 30 cm from each other. On each sprinkler boom, two nozzles were fixed at a distance of 1.5 m from each other. A range of 20–140 mm/h rainfall intensity can be achieved by changing the hydrostatic pressure by moving the valve system horizontally. The mean drop size of the rainfall simulator was 1.8 mm, and the kinetic energy of the rainfall simulator was approximately 75% that of natural rainfall [19]. The effective rainfall area of the simulator was 3 m×3 m, and rainfall uniformity was >80%. The side-sprinkling rainfall simulation system was used for outdoor rainfall simulation. Rainfall devices included the rainfall system and the water supply system. The rainfall system consisted of two single rainfall vertical brackets. A rainfall vertical bracket includes the side sprinkler nozzle, nozzle stents, and pressure-control section. The side-sprinkler nozzle was made up of a nozzle body, steam breaker, and outflow orifice. The nozzle was installed on the rainfall vertical bracket and fixed by a tripod. Each nozzle was 6 m above the ground, and the raindrop spray height was 1.5 m as it sprayed out of the outflow orifice. Therefore, the height from which raindrops reached the ground was 7.5 m, and the effective rainfall area was 5×7 m^2^. The simulated rainfall pattern was created by opposing sprays from these two single rainfall vertical brackets, forming a superimposed rainfall area. The kinetic energy of the side-sprinkling rainfall simulator was similar to that of natural rainfall, and rainfall uniformity was >80%. Supply pressure was controlled by a pressure gauge, and rainfall intensity was controlled mainly by adjusting the supply pressure and bore diameter of the outflow orifice. Rainfall intensity could be controlled over a range of 30–140 mm/h.

### Determination of throughfall

**Figure 1:**
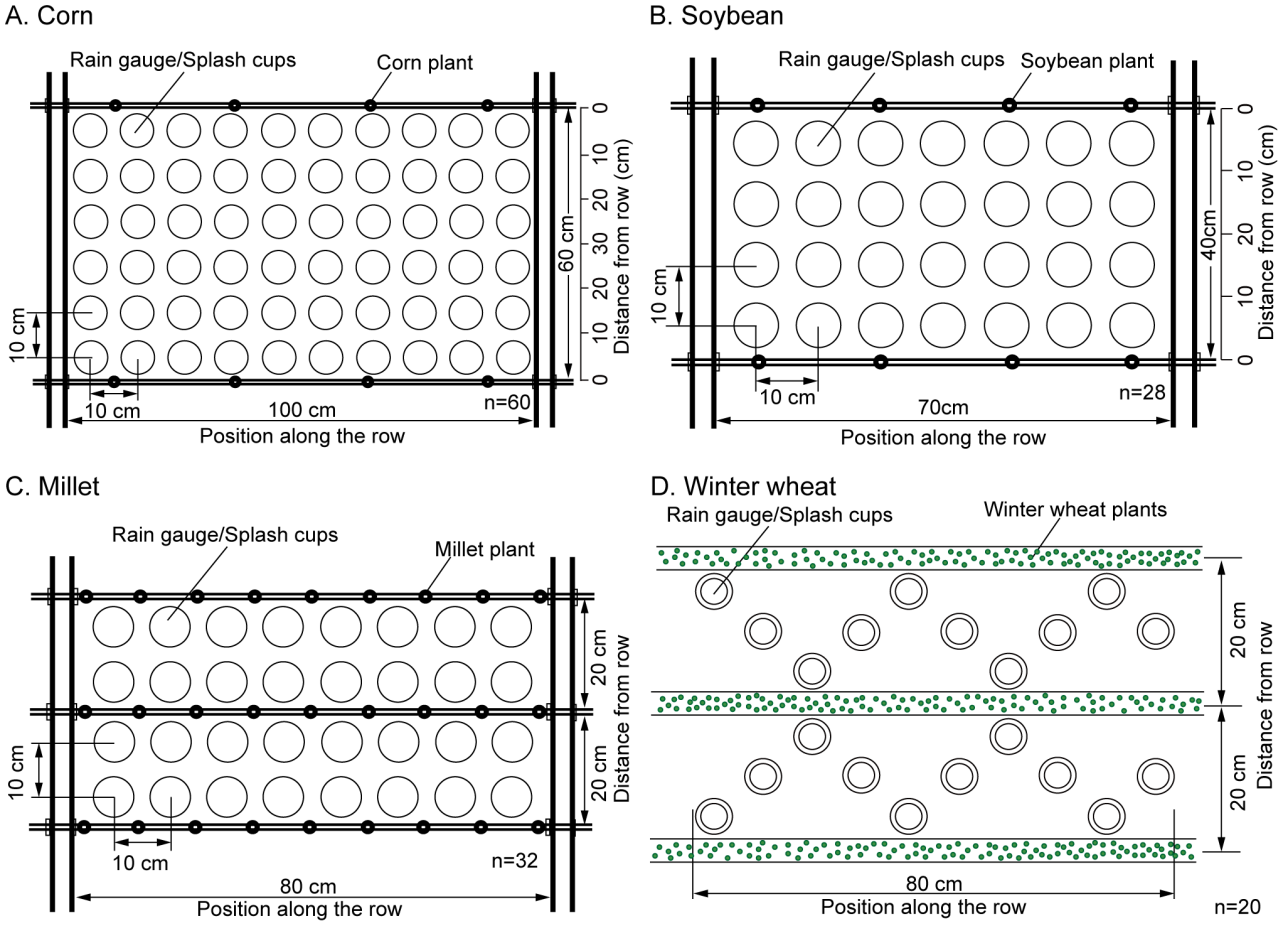
Schematic diagram of measurement for throughfall and splash detachment under crop canopy.

On each sampling date, experimental crops were cut off at the ground, quickly moved indoors, and fixed under the rainfall simulator. Each crop was arranged according to its row spacing (corn, 25×60 cm; soybeans, 20×40 cm; millet, 10×20 cm) and fixed upright in the steel frame. Then the rain gauges were located under the crop canopies in a matrix pattern (Figure 1). Each rain gauge had a diameter of 5.5 cm and a height of 7 cm. After 30 minutes of simulated rainfall, rainwater in each rain gauge was collected and calculated, and the throughfall amount and throughfall intensity of each point were calculated. Finally, the crops were removed, glasses were situated in their place, and rainfall was continued for another 30 minutes under the same rainfall intensities. The design rainfall intensities were 40 mm/h and 80 mm/h according to the characteristics of local storms which are concentrated in summer and autumn.

**Figure 2:**
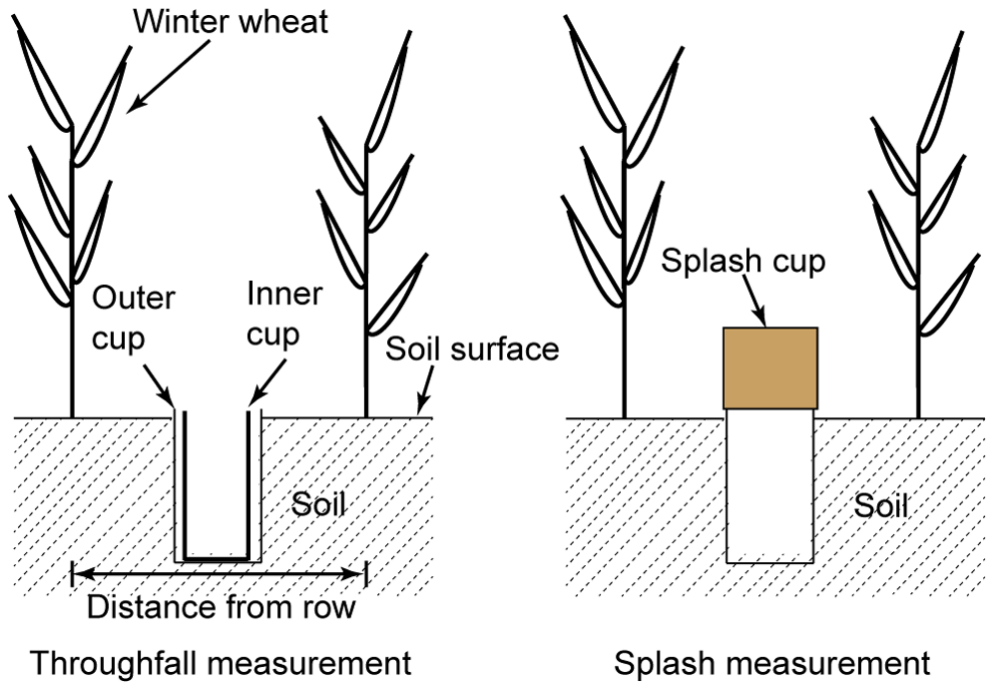
Measurement for throughfall and splash detachment under wheat canopy.

Due to tilling, throughfall for winter wheat could not be simulated indoors, and therefore these experiments were carried out in the field. Rain gauges were placed in an “S” pattern under the crop canopies, which were formed by two rows of crops, at a rate of 10 gauges per row (Figure 1d). Meanwhile, 20 rain gauges were also located in a bare field close to the row of wheat plants to determine rainfall amount. Each rain gauge was placed using the method of inner and outer sleeves. The outer cylinders were embedded in soil (diameter of 6.5 cm, height of 7.5 cm), and the top cylinder was embedded flush with the ground. When measuring, rain gauges were placed in the outer cylinders as the inner sleeve collected rain water (Figure 2).

### Determination of splash detachment rate

The splash detachment rate was determined using the method of splash cups [14]. Splash cups were used to test splash erosion under the canopy at the same place after every throughfall test. Each splash cup was 5 cm high with a diameter of 7 cm and permeable holes evenly located in the bottom of the cup. Each soil sample was sifted using 5-mm sieves and then oven-dried at 105°C until the weight became constant. The splash cup base was paved with filter papers, then filled with sieved soil, and then weighed. Similarly to the throughfall determination, splash cups were placed under the crop canopies. After 30 minutes of rainfall at rainfall intensity of 40 mm/h or 80 mm/h, the splash cups were taken out and oven-dried at 105°C to a constant weight. The difference in the weight of soil in the splash cup before and after artificial rainfall was defined as the splash amount per cup. The splash erosion amount per unit area and per unit time (the splash detachment rate, SDR) was calculated according to the diameter of the splash cup and the rainfall duration. The splash detachment rate on bare soil was determined using the same method. The rainfall amount and intensity were also determined using a gauge located as shown in Figure 1. All these steps were followed to calculate the intensities of throughfall, rainfall, and splash under design rainfall rates of 40 mm h^−1^ and 80 mm h^−1^.

### Measurement of Leaf Area Index

Splash erosion yields in the bare field were determined using the same method. Furthermore, at the end of each stage, the leaf areas of corn, millet, and winter wheat were determined by the method of length-to-width ratio, and total leaf area was determined according to the following formula:
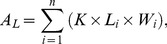
(1)where *A*_L_ is the total area of each plant (cm^2^), *K* is a modification coefficient (corn 0.75, millet and winter wheat 0.85), *L*_i_ is the length of the *i*-th leaf (cm), *W*_i_ is the width at the widest point of the *i*-th leaf (cm), and *n* is the number of leaves on a plant.

The leaf area of soybeans was determined by the method of special leaf weight, and total leaf area was determined using the following formula:
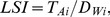
(2)

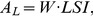
(3)where *LSI* is the special leaf area (cm^2^/g), *T*_Ai_ is a unit of leaf area in some growth period (cm^2^), *D*_Wi_ is the dry weight of a unit of leaf area in some growth period (g), *A*_L_ is leaf area (cm^2^), and *W* is the dry weight of leaves of the test crops (g).

The leaf area index (LAI) was calculated as the green leaf area per unit ground area in broadleaf canopies [20].

## Results and Analysis

### Influence of the crops on splash detachment rate

The characteristics of splash detachment rate and throughfall intensity under the four crop canopies during the whole growth period are given in Table 2. Under experimental conditions, the mean splash detachment rate under the four crop canopies during the whole growth period was 390.12 g/m^2^·h, which was lower by 67.81% than on bare land, suggesting that crops can intensively inhibit splash erosion. This ability indicated that the initial phase (raindrop erosion process) was very weak when water erosion occurred on cultivated land with crop cover. The mean splash detachment rate under the corn canopy during its growth period was 380.43 g/m^2^·h (68.32% less than bare land), under soybeans, 489.56 g/m^2^·h (60.76% less than bare land), under millet, 627.84 g/m^2^·h (17.94% less than bare land), and under winter wheat, 62.66 g/m^2^·h, the lowest value and 94.75% less than bare land. These values show that splash erosion by confluence on the soil surface differs by crop variety and depends on rainfall intensity, throughfall intensity, and crop growth status.

**Table 2:** Splash detachment and throughfall under crop canopy in different growth stage.

Rainfall intensity (mm/h)	Crop type	Observation date	Average rainfall intensity up the canopy (mm/h)	Average throughfall intensity under the canopy (mm/h)	Ratio of throughfall amount to the total rainfall (%)	Splash detachment rate on bare soil (g/m^2^·h)	Splash detachment under crop canopy	
							Average splash detachment rate under crop canopy (g/m^2^·h)	Coefficient of dispersion
40	Corn	2009/7/10∼8/17	40.37	29.19	72.40	427.97	202.48	1.42
	Soybean	2007/7/30∼8/28	40.49	32.02	79.07	458.09	172.82	1.35
	Millet	2008/5/25∼6/26	40.36	34.35	85.87	423.80	325.57	1.15
	Winter wheat	2008/3/15∼5/2	39.89	31.52	78.80	430.20	41.43	1.26
80	Corn	2009/7/10∼8/17	80.24	57.66	71.82	1973.57	558.39	1.69
	Soybean	2007/7/30∼8/28	80.58	66.12	82.05	2037.08	806.31	1.27
	Millet	2008/5/25∼6/26	80.59	59.94	74.93	1988.20	930.11	0.91
	Winter wheat	2008/3/15∼5/2	80.46	66.13	82.66	1957.86	83.90	0.97

Table 2 also shows that the splash detachment rate under high rainfall intensity (80 mm/h) was two to five times higher than under low rainfall intensity (40 mm/h). The effect of rainfall intensity on splash erosion was greatest under the soybean canopy, where splash erosion was 4.67 times higher under 80 mm/h rainfall intensity than under 40 mm/h rainfall intensity. The change in splash detachment rate under winter wheat at the two rainfall intensities was the least, 2.03 times. The ability of crops to inhibit splash erosion increased by 13% on average as rainfall intensity increased. The ability of corn, millet, and winter wheat to inhibit splash erosion rose by 19.02%, 30.04%, and 5.34% respectively as rainfall intensity increased, while the ability of soybeans to inhibit splash erosion decreased by 2% on average as rainfall intensity increased.

**Figure 3:**
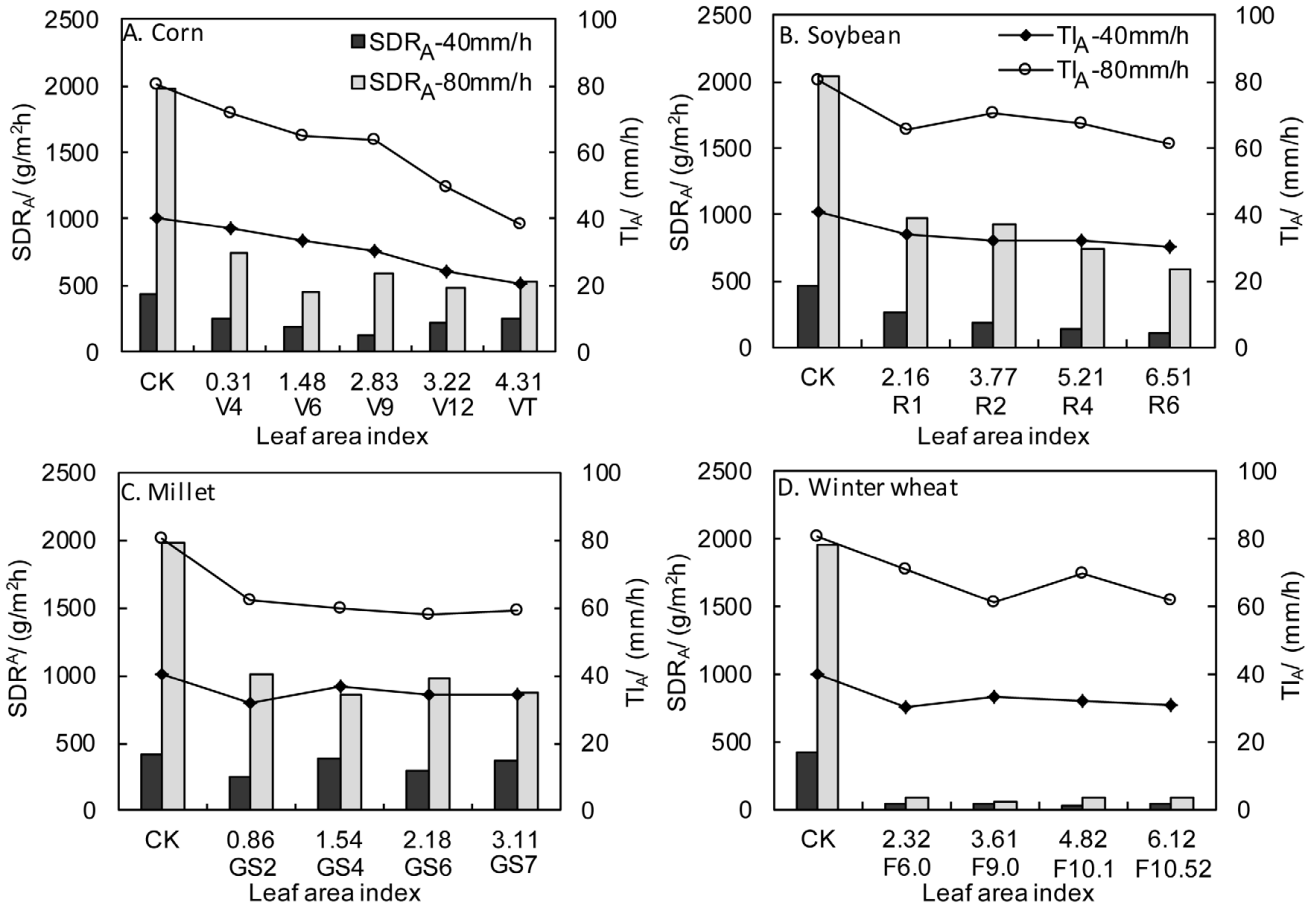
Relationship between throughfall and average splash detachment rate under crop canopy.

Figure 3 shows the relationship between throughfall intensity and splash detachment rate under different crop canopies at the testing stage. Clearly, there was a significant relationship between throughfall intensity and splash detachment rate under the crop canopies. Under low rainfall intensity (40 mm/h), except for the R4 stage of soybeans (LAI = 5.21), splash detachment rate increased with rainfall intensity. However, this relationship became complex when rainfall intensity was high (80 mm/h), depending on plant height during different growth periods and the ability of branches and leaves to bear raindrop impact. At low rainfall intensity, because of fewer raindrop impacts and longer time to form large raindrops from rain convergence on leaves, large raindrops contributed less to splash erosion, and the regularity became obvious. However, this phenomenon was reversed at high rainfall intensity, and therefore the regularity was not obvious.

Figure 3 also shows that the leaf area index increased and the ability of crops to inhibit splash erosion also increased with crop growth. The effects of corn on splash erosion changed significantly with crop growth, but the variation was random (Figure 3a). Under 40 mm/h rainfall intensity, splash detachment rates fluctuated more widely under corn canopies in the whole growth season, with the splash detachment rate being low in the V6 and V9 stages (LAI = 1.48–2.83), then increasing in the V12 stage (LAI = 3.22); splash erosion in the VT stage (LAI = 4.31) was very close to that in the V4 stage (LAI = 0.31), although the leaf area index was larger. Under 80 mm/h rainfall intensity, the splash detachment rate also fluctuated irregularly. However, the splash detachment rate in the VT stage decreased by 30% compared to the V4 stage, which indicated that the corn canopy could significantly reduce the splash detachment rate, but there was no regularity in its inhibition of splash erosion.

As for soybeans (Figure 3b), the mean splash detachment rate under the soybean canopy tended to decrease as the soybeans grew under both rainfall intensities, and the splash detachment rate decreased by 59.68% at 40 mm/h rainfall intensity and 40.18% at 80 mm/h rainfall intensity from the R1 stage (LAI = 2.16) to the R6 stage (LAI = 6.51), which indicated that the inhibition effects on splash erosion increased more regularly as the soybeans grew. Rainfall intensity had a marked effect on splash erosion. Splash erosion in the R1 stage at 80 mm/h rainfall intensity was 3.69 times greater than at 40 mm/h rainfall intensity, but this differences increased to 5.48 times in the R6 stage, when the splash detachment rate at high rainfall intensity was far higher than at low rainfall intensity, and this differences increased further as the soybeans grew. Maybe this phenomenon occurred because rainfall kinetic energy at high rainfall intensity could increase the bare areas between rows and reduce energy dissipation by the canopy. Compared to corn, splash erosion was lower under the soybean canopy at rainfall intensity of 40 mm/h, suggesting that the inhibition ability of the soybean canopy on splash erosion was greater than that of the corn canopy under low rainfall intensity, but less under high rainfall intensity. Under 80 mm/h rainfall intensity, rainfall kinetic energy caused more throughfall between soybean rows and increased the bare area between rows compared to corn. This increased bare-field splash erosion, which caused a higher splash detachment rate under the soybean canopy compared to corn under high rainfall intensity. However, under 40 mm/h rainfall intensity, lower rainfall kinetic energy affected the soybean canopy less, which together with the short and dense form of the soybean canopy and its better surface coverage, which greatly reduced rainfall kinetic energy, reduced the effects of soybeans on splash erosion compared to corn.

Splash erosion changed under the millet canopy as the plants grew, with significant fluctuations. Under 40 mm/h rainfall intensity, splash erosion fluctuated greatly and increased as the plants grew, but decreased when rainfall intensity was 80 mm/h (Figure 3c), which indicates that the inhibition effect of the millet canopy on splash erosion varied significantly at different rainfall intensities. The inhibition effects on splash erosion were increased by 53.22% compared with bare land under high rainfall intensity, but were reduced by 30% under 40 mm/h rainfall intensity, which indicates that the inhibition effect of millet on splash erosion increased at higher rainfall intensity.

Splash erosion changed under the winter wheat canopy as the plants grew, with significant fluctuations (Figure 3d). Under 40 mm/h rainfall intensity, splash detachment rate under the winter wheat canopy varied from 35.58 g/m^2^·h to 50.39 g/m^2^·h. Under 80 mm/h rainfall intensity, detachment rate varied from 65.71 g/m^2^·h to 93.25 g/m^2^·h. This suggests that the inhibition effect of winter wheat on splash erosion varies with rainfall intensity. Under 40 mm/h rainfall intensity, splash erosion yield decreased by 90% compared to bare land, but by 96% under 80 mm/h rainfall intensity compared to bare land, suggesting that the inhibition effect of winter wheat on splash erosion increased with rainfall intensity.

The inhibition effects of corn, millet, and winter wheat on splash erosion were greater under 80 mm/h rainfall intensity than under 40 mm/h rainfall intensity. The inhibition effect on splash erosion was increased by 30% under 80 mm/h rainfall intensity over that at 40 mm/h rainfall intensity, the biggest jump among the four crops, while erosion was least under the winter wheat canopy and increased by only 5%. Inhibition of splash erosion by soybeans differed little between the two rainfall intensities. The inhibition ability of the four crops on splash erosion was in the order of winter wheat > corn > soybeans > millet. Because of its short plants and dense canopy, winter wheat could inhibit splash erosion markedly and thus protect the soil effectively.

A regression analysis of the effects of leaf area index and throughfall intensity on splash detachment rate during the whole growth stage was carried out, with the results shown in Table 3. The relationships among average splash detachment rate, LAI, and average throughfall intensity were highly significant (P<0.01). Therefore, splash detachment rates of different crops at different growth stages can be estimated using this regression equation.

**Table 3:** Regression about the average splash detachment rate under crop canopies.

Crop types	Regression formula	R^2^	F value
Corn	*SDR*_A_ = 47.618*LAI*+11.659*TI*_A_ −241.592	0.812	15.131^**^
Soybean	*SDR*_A_ = −49.046*LAI*+18.496 *TI*_A_ −201.658	0.982	133.732^**^
Millet	*SDR*_A_ = 13.274*LAI*+23.593 *TI*_A_ −509.926	0.976	103.160^**^
Winter wheat	*SDR*_A_ = 2.470*LAI*+1.227 *TI*_A_ −7.660	0.884	19.037^**^

where, *SDR*_A_ was average splash detachment rate under crop canopy, g/m^2^·h; LAI was leaf area index; *TI*_A_ was average throughfall intensity under crop canopy, mm/h.

### Spatial distribution of splash erosion

**Figure 4:**
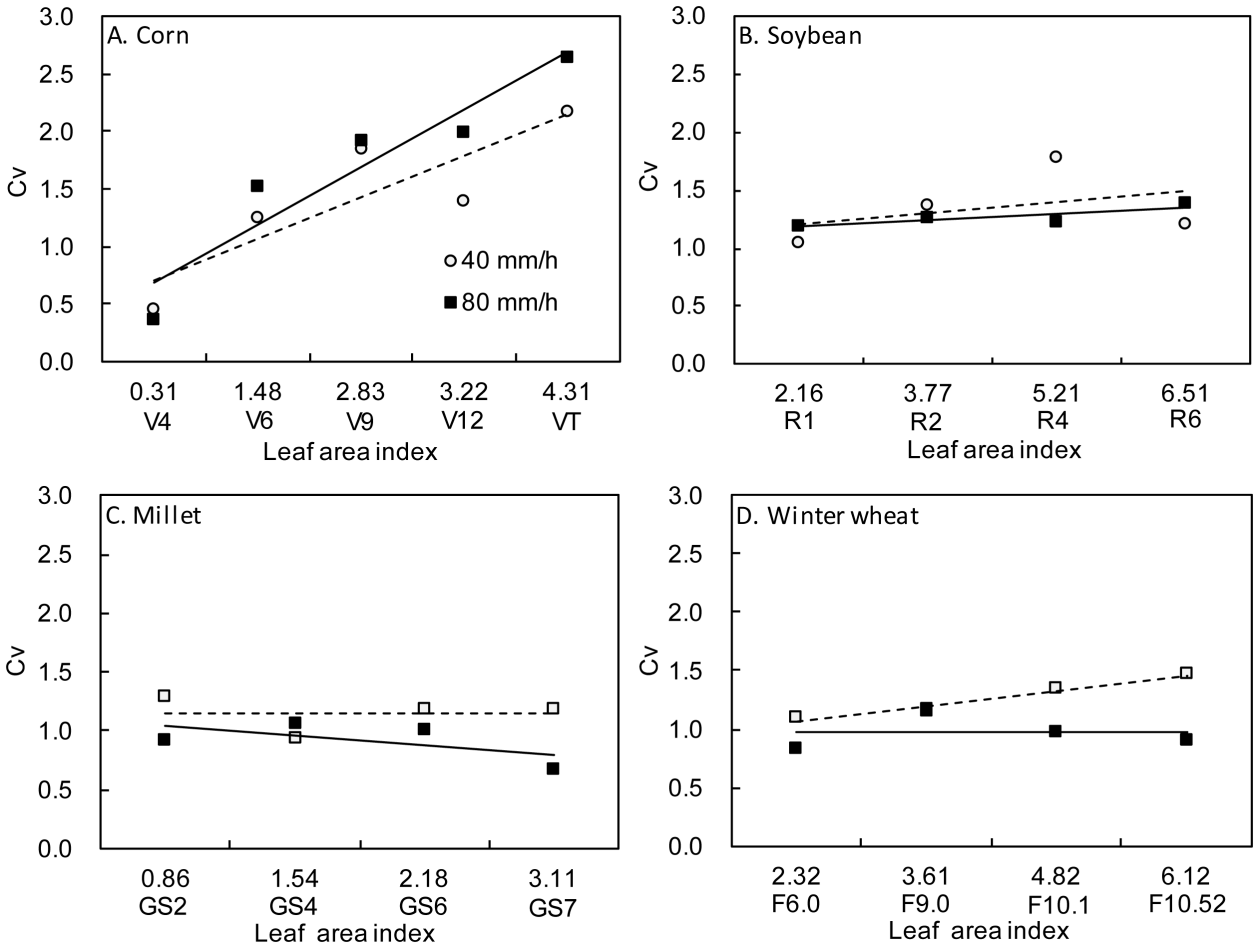
Relationship between Cv of splash detachment and crop growth.

The coefficient of dispersion is an index which describes the degree of dispersion of a sample. According to statistical data, the degree of dispersion of point splash erosion yield increased with crop growth (Figure 4). Clearly, the spatial distribution of splash erosion was relatively uniform over cultivated land at early crop growth stages. However, the distribution was not uniform in middle and later growth stages.

The *CV* of splash detachment rate under corn canopies increased from 0.37 at the V4 stage to 2.65 at the VT stage, suggesting that the spatial distribution of splash detachment rate became less uniform as the corn grew. The *CV* of splash detachment rate under soybean canopies increased from approximately 1.0 at the R1 stage to approximately 1.7 at the R4 stage; this non-uniformity increased with soybean growth, but decreased at the R6 stage. The *CV* of splash detachment rate under millet canopies ranged from 0.67 to 1.28 during the whole growth period, and this variation was not regular with crop growth, suggesting that the variability of splash detachment rate under millet canopies was low and that the spatial distribution of splash detachment rate differed only slightly under both rainfall intensities during the whole growth period compared to corn and soybeans. The *CV* of splash detachment rate under winter wheat canopies ranged from 0.83 to 1.47 during the whole growth period, suggesting that the spatial distribution uniformity of splash detachment rate under winter wheat canopies was lower. The *CV* increased from 1.09 at the Feekes 6.0 stage to 1.47 at the Feekes 10.52 stage under 40 mm/h rainfall intensity, whereas the variation was irregular under 80 mm/h rainfall intensity and the differences were small. This indicated that the non-uniformity of splash detachment rate increased with winter wheat growth under low rainfall intensity, but that non-uniformity was stable and differences were small under high rainfall intensity.

Let us define the 0–20 cm band nearest the corn plants as the region directly under the canopy (the 0–20 cm and 20–0 cm bands in Figure 1a) and the 20–30 cm band between the plants as the region in the central row position (the 20–30–20 cm band in Figure 1a). The area between two rows of soybean plants is divided into a 0–10 cm band nearest the soybean plants (the region directly under the canopy, the 0–10 cm and 10–0 cm bands in Figure 1b) and a 10–20 cm band (the region in the central row position, the 10–20–10 cm band in Figure 1b). If the *SDR*_CR_/*SDR*_DUC_ ratio were reflected in the concentration and spatial distribution of splash erosion, it could be suspected that splash erosion occurs mainly between crop rows and this phenomenon becomes more obvious as crops grow, as shown in Table 4.

**Table 4:** Distribution of splash erosion under crop canopy in whole growth stage.

	Corn						Soybean					
Rainfall intensity (mm/h)	Growth stage	LAI	Average splash detachment rate under canopy(*SDR*_A_, g m^−2^h^−1^)	Splash detachment rate in the region direct under the canopy (*SDR*_DUC_, g m^−2^h^−1^)	Splash detachment rate in the central row position (*SDR*_CR_, g m^−2^h^−1^)	*SDR*_CR_ */SDR*_DUC_	Growth stage	LAI	Average splash detachment rate under canopy(*SDR*_A_, g m^−2^h^−1^)	Splash detachment rate in the region direct under the canopy (*SDR*_DUC_, g m^−2^h^−1^)	Splash detachment rate in the central row position (*SDR*_CR_, g m^−2^h^−1^)	*SDR*_CR_ */SDR*_DUC_
40	V4	0.31	247.77	252.70	237.92	0.94	R1	2.16	264.56	208.53	320.59	1.54
	V6	1.48	183.41	187.47	171.33	0.91	R2	3.77	187.38	69.39	305.38	4.40
	V9	2.83	125.29	114.97	145.91	1.27	R4	5.21	132.65	84.23	181.08	2.15
	V12	3.22	211.87	156.99	321.62	2.05	R6	6.51	106.68	93.14	120.22	1.29
	VT	4.31	244.05	219.06	294.04	1.34						
	Average	2.43	202.48	186.24	234.16	1.26	Average	4.41	172.82	113.82	231.82	2.34
80	V4	0.31	748.33	768.75	707.48	0.92	R1	2.16	976.99	623.75	1330.24	2.13
	V6	1.48	452.05	425.13	505.88	1.19	R2	3.77	919.29	392.21	1446.38	3.69
	V9	2.83	592.06	543.93	688.31	1.27	R4	5.21	744.53	474.95	1014.10	2.14
	V12	3.22	478.74	391.52	653.17	1.67	R6	6.51	584.42	161.78	1007.05	6.22
	VT	4.31	520.76	480.52	601.22	1.25						
	Average	2.43	558.39	521.97	631.21	1.21	Average	4.41	806.31	413.17	1199.44	3.55

Splash detachment rate in the central row position (20–30 cm band) increased by 28.11% compared with the regions directly under the canopy (0–20 cm band) during the whole period of corn growth. The *SDR*_CR_/*SDR*_DUC_ ratio increased from 0.9 to more than 2, suggesting that splash erosion under canopies tends to occur in central row positions as corn grows, with the largest observed values at the V12 stage. The splash detachment rate slightly decreased at the VT stage, and the *SDR*_CR_/*SDR*_DUC_ ratio decreased by approximately 0.4–0.7 compared to the V12 stage. Splash erosion was focused mainly in the 0–20 cm band at the V4 stage of corn. Under 40 mm/h and 80 mm/h rainfall intensities, average splash detachment rates were 1.06 and 1.09 times higher than in the 20–30 cm band, but the difference was small. This indicated that the distribution of splash erosion under the corn canopy was uniform when the corn plants were small. As the corn grew, the corn canopies changed, and therefore the splash concentration moved from the 0–20 cm band to the 20–30 cm band. It reached its maximum at the V12 stage, when average splash detachment rates were 2.05 and 1.67 times higher than in the 0–20 cm band under 40 mm/h and 80 mm/h rainfall intensity respectively. The above discussion shows that splash erosion under corn canopies is highly concentrated in the central row position, with splash erosion yield in the central row position accounting for more than half the total splash erosion yield under canopies. When corn was in the VT stage, the corn leaves grew to their greatest extent, and canopy breadth also reached a maximum. Under these conditions, large water drops formed and fell from the leaf edge and apex, and the concentration moved from the 20–30 cm band to the 0–20 cm band, leading to greater splash erosion yield in some positions (10–20 cm band) near the between-row space. Under 40 mm/h and 80 mm/h rainfall intensities, average splash detachment rates in the 20–30 cm band were 1.34 and 1.25 times higher than in the 0–20 cm band in the VT stage. This indicated that splash erosion yield was concentrated mainly in the 20–30 cm band and in some positions in the 10–20 cm band, while the splash erosion yield in the 0–10 cm band nearest the corn plants gradually decreased with crop growth.

Unlike corn, the splash detachment rate in the 10–20 cm band was 194.49% higher than in the 0–10 cm band during soybean growth. Under 40 mm/h rainfall intensity, the *SDR*_CR_/*SDR*_DUC_ ratio expanded its range from 1.4 to 2.2, and from 2.1 to 6.3 under 80 mm/h rainfall intensity. This suggests that splash erosion yield was concentrated mainly in the central row position and that this concentration increased greatly at high rainfall intensity. The average splash detachment rate in the 10–20 cm band was 1.54 times higher than in the 0–10 cm band in the R1 stage. From the R1 to the R2 stage, splash erosion was mostly concentrated in the central row area and appeared in the form of a zonal distribution. Afterwards, in the R4 stage, although the maximum splash detachment rate data points still occurred in the 10–20 cm band, the probability of occurrence of extreme splash detachment rates was decreased, in particular that of the higher splash detachment rates shown as a dotted distribution in the R4 and R6 stages. The splash erosion yield at these high-concentration points accounted for much of the erosion, 48.36% at the R4 stage and 37.96% at the R6 stage of total splash erosion yield. This indicated that the splash erosion yield gradually decreased with soybean growth, but that the splash erosion yield remained mostly concentrated at a few points in the central row position with a large proportion of total splash erosion.

Considering the above analysis, the splash detachment and its spatial distribution under crop canopy were related closely to the big raindrops which make up the throughfall under crop canopy. The big raindrops form from the leaf edge and apex are a source of kinetic energy which can bring about erosion, and leading to uneven distribution of splash detachment. The leaf shapes are different with different crops, and leading to the different big raindrops form ability. Corn, millet and winter wheat are all gramineous plants with long and narrow leaves, but the leaves of corn are relatively large with waving leaf edge. The leaves could deflexed with the corn growth, and caused rain water on the leaves flowed to apex and the hollow of leaf edge. Thus forming considerable big raindrops under the canopy, and facilitating the splash erosion. Soybean is a kind of leguminous plant with wide and soft leaves. The leaves were apt to bend down when they undertake the rainfall, thus form considerable big raindrops from the leaf apex. Therefore, throughfall under corn and soybean canopies had higher numbers of big raindrops, which can increased the splash detachment. Besides that, there are great differences between different crops height. Corn could reach a maximum height of 2.2 m in the observation period, while 0.9∼1.2 m of max plant height in soybean, millet and winter wheat growth period. It has strong kinetic energy when big drops falling from higher corn leaves, and increasing splash erosion sharply. It indicated that, high stalk crops such as corn have serious splash erosion under the canopy compare other crops which have lower canopy height.

## Conclusions and Discussion

Crop canopies can effectively reduce rainfall kinetic energy and protect soil surfaces from raindrop impact, thus inhibiting splash erosion. However, for different crop types and growth status, the effects on splash erosion were different. Results indicated that the average splash detachment rate under crop canopies was 390.12 g/m^2^·h, which represented a decrease of 67.81% compared with bare land. Crop coverage had some effect on reducing raindrop impact and preventing splash erosion, and different types of crops had different effects on reducing splash erosion. In this study, winter wheat had the strongest inhibition effects on splash erosion, followed by corn and soybeans, with millet the lowest. As the crops grew, the splash erosion yield under their canopies decreased, which was in accordance with the results reported by Miao [21], but different from those of Morgan [13]. Morgan conducted experiments on splash erosion under corn and soybean canopies and concluded that splash erosion increased with the height of the corn canopy, but the reverse occurred for soybeans. On the contrary, in this study, the average splash erosion yield under corn canopies decreased as the plants grew, while splash erosion at some points between corn rows was far greater than on bare land. In addition, rainfall intensity had a significant effect on inhibiting splash erosion; the splash erosion inhibition ability of crops was 13% greater under 80 mm/h rainfall intensity than under 40 mm/h rainfall intensity.

Rainfall was intercepted by crop canopies, was divided into three parts (throughfall, stemflow, and canopy interception), and then fell into the soil surface or was dissipated [8]. The effects of crops on splash erosion were influenced mainly by throughfall intensity, raindrop diameter distribution, and energy variations [12]. Throughfall intensity under crop canopies was closely related to splash detachment rate, which indicated that splash detachment rate increased with throughfall intensity. Under 40 mm/h rainfall intensity, the relationship between splash detachment rate and throughfall intensity was more significant, while the relationship became complex under 80 mm/h rainfall intensity because of other factors.

The spatial distribution of splash erosion under crop canopies became less uniform between rows, with splash erosion evidently tending to occur intensively in central row positions. Although throughfall under crop canopies decreased with crop growth, it tended to converge in central rows, which caused marked splash erosion and created a concentration of splash erosion in the central row position. This was consistent with reports by Armstrong and Mitchell [8] and Quinn and Laflen [9]. The latter studied the effects of throughfall on splash erosion under crop canopies, made a comparison to data from the USLE model, and concluded that raindrops from the leaf margin and apex were an important factor in soil erosion. Armstrong and Mitchell [8] believed that large raindrops formed by rainfall convergence from crop canopies and focusing on a small impact plot would cause higher soil loss under canopies. Therefore, large throughfall raindrops formed from raindrops on the leaf margin and apex were an important kinetic energy source for splash erosion occurrence and distribution. However, this paper did not include a quantitative analysis of the relationship between large throughfalling raindrops and splash erosion occurrence and distribution. The results showed significant linear relationships among splash erosion, leaf area index, and throughfall intensity, suggesting that crop growth made the relationship between soil surface splash erosion and rainfall more random and complex.

Based on the above analysis, under suitable local conditions, rational close planting and make full use of the population dominance will be a good way to reduce splash erosion while boost crop yields. The compact crop types of corn and sorghum would be selected to increase the LAI, thus reduce throughfall by crisscrossed leaves. Furthermore, corn intercropping with other crops such as soybean and potato could be considered to increase the coverage between the rows of high stalk crops thereby reduces splash erosion.
